# Degradation and Recondensation of Metal Oxide Nanoparticles in Laminar Premixed Flames

**DOI:** 10.3390/nano14121047

**Published:** 2024-06-18

**Authors:** Nadine May, Werner Baumann, Manuela Hauser, Zhiyao Yin, Klaus Peter Geigle, Dieter Stapf

**Affiliations:** 1 Institute for Technical Chemistry, Karlsruhe Institute of Technology, 76344 Eggenstein-Leopoldshafen, Germany; werner.baumann@kit.edu (W.B.); manuela.hauser@kit.edu (M.H.); dieter.stapf@kit.edu (D.S.); 2 Institute of Combustion Technology, German Aerospace Center (DLR), Pfaffenwaldring 38-40, 70569 Stuttgart, Germany; zhiyao.yin@dlr.de (Z.Y.); klauspeter.geigle@dlr.de (K.P.G.)

**Keywords:** nano-enabled products, volatility of metal oxides, CARS temperature measurement, thermal degradation of nanoparticles

## Abstract

The behavior of technical nanoparticles at high temperatures was measured systematically to detect morphology changes under conditions relevant to the thermal treatment of end-of-life products containing engineered nanomaterials. The focus of this paper is on laboratory experiments, where we used a Bunsen-type burner to add titania and ceria particles to a laminar premixed flame. To evaluate the influence of temperature on particle size distributions, we used SMPS, ELPI and TEM analyses. To measure the temperature profile of the flame, we used coherent anti-Stokes Raman spectroscopy (CARS). The comprehensible data records show high temperatures by measurement and equilibrium calculation for different stoichiometries and argon admixtures. With this, we show that all technical metal oxide nanoparticle agglomerates investigated reform in flames at high temperatures. The originally large agglomerates of titania and ceria build very small nanoparticles (<10 nm/“peak 2”) at starting temperatures of <2200 K and <1475 K, respectively (ceria: T_melt_ = 2773 K, T_boil_ = 3873 K/titania: T_melt_ = 2116 K, T_boil_ = 3245 K). Since the maximum flame temperatures are below the evaporation temperature of titania and ceria, enhanced vaporization of titania and ceria in the chemically reacting flame is assumed.

## 1. Introduction

The use of engineered nanomaterials (ENMs) in everyday products is constantly growing. Because of their nanoscale, these particles have fascinating properties in contrast to bulk material. For example, silicon dioxide is used in tires to reduce abrasion. Silver nanoparticles have antibacterial properties and are used in medical products and sportswear. Nanoscale cerium dioxide is used as a diesel fuel additive to reduce particulate matter emissions and fuel consumption in diesel engines. Nanoscale titanium dioxide is added to sunscreen because of its protective effect against UV radiation.

Nanoparticles are often used as additives and incorporated particularly in thermoplastics. There, they act, for example, as fillers, color pigments or flame retardants and enhance the mechanical, optical or thermal properties of thermoplastics strongly.

At the end of life (EoL), these products must also be safely and properly recycled or disposed of. This so-called “nanowaste” requires special attention as its processing, recycling and disposal may release nanoparticles, making it a possible hazard for humans and the environment [1,2].

Industrially produced nanoparticles with the highest production amounts are carbon black, titanium dioxide, and silicon dioxide (silica), which are generally produced by flame synthesis. However, there are no reliable figures regarding the production amounts of ENM in Germany, Europe or worldwide as there is no legal obligation to report them, with some exceptions in France, Belgium, Denmark, Norway and Sweden [3]. To fill this gap, some authors [4,5,6,7,8] have conducted surveys of experts in the industry, who in turn have estimated the production amounts. A comparison of these figures can be found in Table 1, which also includes CeO_2_ and carbon nanotubes (CNTs) and the mass-produced ENM.

The table shows that the individual estimates differ significantly, even though they took place at approximately the same time. The more recent the study, the higher the estimated production volumes, which correlates with the general growth of the nanomaterials market.

The same data gap exists concerning nano-enabled products. In general, there is very little information about which products contain nanomaterials, as there are only a few areas, such as cosmetics, food packaging and biocidal substances, where specific legislation exists in Germany and the EU [10]. So far, only France, Denmark, Norway, Belgium, Italy and Sweden have registers in which products containing nanomaterials must be recorded [11]. Within the category of metal oxide nanomaterials, titania is by far the most used nano additive [12].

Titanium dioxide is mainly used as a white pigment in a wide range of products, and globally, several million tons of titanium dioxide are processed annually in plastics, coatings, paints, food, cosmetic products and pharmaceuticals [13].

An admixture of nanoparticulate additives can have an impact on the entire process chain, from production to disposal. According to a German study by Conversio Market & Strategy GmbH [13], about 142 kt of TiO_2_ was incorporated into 14.4 million t of plastic products in Germany in 2017. Thus, on average, the TiO_2_ content in plastics is about 1.0 wt.-%, with ⅔ of the TiO_2_ amount used in plastic products in the packaging and construction sectors. Particularly high TiO_2_ contents are present in white plastic profiles, such as window frames, with a TiO_2_ content of 3 to 5%.

The unclear data situation regarding production quantities [14] also makes it difficult to predict the possible amounts of nanomaterials released into the environment based on a life cycle analysis.

There are many potential pathways for the release of nanomaterials along their life cycle, whether during production, use, or recycling [15,16]. If recycling is possible, depending on the kind of product, it is always associated with downcycling. Ultimately, in Germany and various other countries, waste is thermally recovered. During this incineration, mineral waste content including ENM is to be converted into harmless ash for disposal or further use as construction material.

For this reason, we previously dealt with the behavior of nanomaterials during thermal utilization, investigated whether nanoparticles can be released in large-scale plants via the gas path, and determined the sinks within these plants for ceria particles [17,18,19]. Cerium dioxide was used as tracer material because it is hardly found in the background of such plants. Therefore, the samples taken were analyzed for cerium quite easily. There are also studies where titanium dioxide has been introduced into incinerators, but since it occurs far more frequently, it is very difficult to distinguish the added titanium dioxide from that in the background, so the results of these studies are subject to significant fluctuations [20].

In order to be able to predict the behavior of nanoparticles in the complex aerosol of combustion plants, fundamental studies are required. To our knowledge, very few studies have dealt with the addition of nanoparticles to flames. In one of these published studies, particles were dosed into flames in powder form and the effects on the particle size distribution were observed [21]. SiO_2_ was added to a methane/oxygen diffusion flame using a brush feeder. With an increasing equivalence ratio, a peak formed at significantly smaller particle sizes, and the distribution was bimodal until the peak disappeared completely at large diameters with further increasing equivalence ratio, where only the new peak could be seen. The processes were confirmed by means of particle dynamics modeling (General Dynamics Equation—GDE), whereby total or partial evaporation with subsequent nucleation, coagulation and surface growth was assumed. In another publication by the authors [22], WO_3_ particles were used with a modal value of the particle size distribution at approx. 700 nm. The temperatures used were also partly above the boiling temperature of WO_3_, which is why the authors used purely physical processes, namely, evaporation, nucleation, coagulation and surface growth, to describe their experimental findings theoretically. As the distance to the burner increased, the number concentration in the new peak decreased and shifted slightly to the right. At a height of approx. 4 cm above the burner, the particle material completely passed into the gas phase, and only at a height of approx. 10 cm above the burner did homogeneous nucleation occur because of supersaturation. The predictions of the model (also GDE) could be confirmed experimentally.

At this point, it must also be briefly pointed out that very small nanoparticles exhibit different physical behavior than the respective bulk material. Buffat and Borel used gold particles to show that the melting temperature changes depending on the particle size [23]. The 2.5 nm gold particles used had a melting temperature that is approx. 1000 °C lower than that of the bulk material. Other authors confirmed this finding [24,25,26] and presented models for calculating these effects. Evaporation at temperatures well below the bulk evaporation temperature has also been observed and interpreted by some authors. In the physical process of NP vaporization, a linear relationship between the vaporization temperature and the reciprocal particle diameter has been reported [24,27,28]. This relationship is also found in other size-dependent thermodynamic quantities, such as the melting temperature, melting enthalpy, melting entropy, Curie temperature and Debye temperature [24]. Because of the significantly different properties of NP compared with the bulk material and the greatly altered thermodynamic behavior, a new interdisciplinary field, nanothermodynamics, has been established [29]. However, the models only predict a strong deviation from the value of the bulk material for various thermodynamic variables below approx. 20 nm.

As described above, TiO_2_ is the most commonly used metal oxide nanomaterial, and studies on the thermal behavior of its particles are not yet available. Therefore, in this study, basic laboratory investigations on the influence of temperature on titania and ceria particles were performed to obtain comprehensible data sets. For this purpose, a well-defined quasi-adiabatic flame stabilized on a Bunsen-type burner was used. Titania and ceria nanoparticles are added to a laminar, premixed flame. With online particle sizing instruments as well as offline image analysis, the influence of flame temperature on the aerosol was investigated to explain and model the experienced phenomena. The results show that titania builds a new particle peak at very small particle sizes at high temperatures. Since the maximum flame temperatures are below the evaporation temperature of titania and the resulting particle size is smaller than the primary particles in the original titania agglomerates, enhanced vaporization of titania in the chemically reacting flame is considered a plausible explanation.

## 2. Materials and Methods

For basic studies at the laboratory scale, the focus was on titania material since it is more relevant to the production amounts than ceria. For the pilot and industrial-scale experiments, we used ceria since it has a very low background in such plants. Therefore, the ceria particles were studied at the laboratory scale as well, but they play a minor role in this paper.

As the flame, we used laminar ethylene–air flames diluted by argon in order to provide a stationary well-defined investigation system for the temperature impact on ENM.

**Figure 1:**
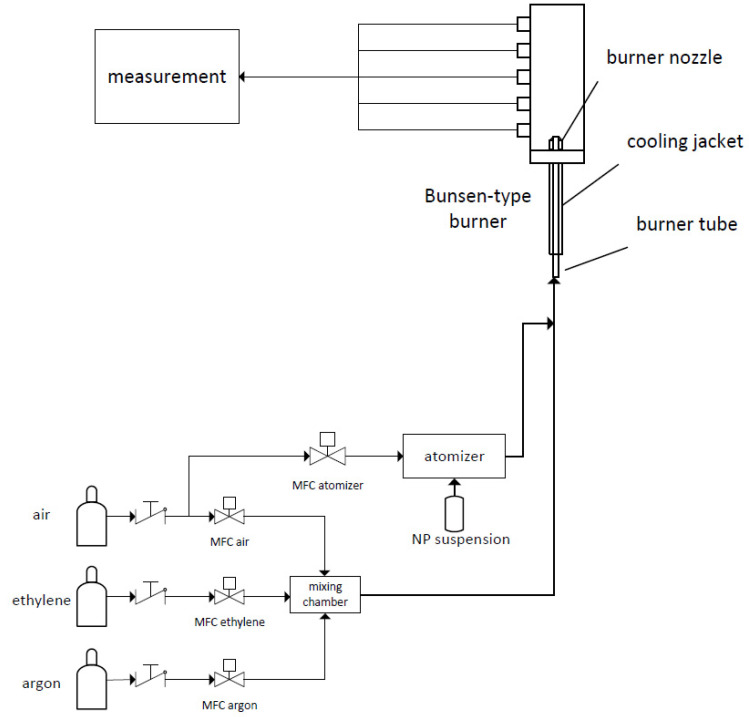
Schematic of the laboratory setup.

This section is divided into 5 parts as follows: a laboratory Bunsen-type burner, the used nanoparticles, aerosol sampling and characterization, equilibrium temperature calculation and temperature measurement via coherent anti-Stokes Raman spectroscopy (CARS). Figure 1 shows the experimental setup.

### 2.1. Laboratory Bunsen-Type Burner

For these investigations, we used a Bunsen-type burner. At the tube outlet, a conical, laminar, premixed flame was stabilized. Nanoparticle suspensions were premixed with the fuel/air mixture and added at the inlet of the burner.

The burner consists of a 70 cm long stainless-steel tube with an inner diameter of 10 mm. The gases were added at the inlet of the burner via 6 mm Swagelok connections. A cooling jacket encloses the gas-carrying tube, through which water flows at a fixed temperature so there is a constant gas inlet temperature at the burner exit.

Mass flow controllers (MFCs) (EL-Flow, Bronkhorst, Ruurlo, The Netherlands) set the volume flow of the needed gases. Synthetic air was used both as an oxidizer for the combustion of ethylene and as a carrier gas for the sprayed nanoparticle suspension. Argon was used to reduce the flame temperature while maintaining the stoichiometry of the combustion. The advantage of the burner design is that the nanoparticles and the fresh gas pass through the flame front together unhindered and no separation can occur, which would be the case with a flat flame burner, for example, where the flame is stabilized on a sintered metal plate. A list of the used settings regarding argon addition and volume flows can be found in Table 2. The total volume flow was fixed to ensure the same cold gas velocity for all used settings. The equivalence ratio Φ is the ratio between the oxygen content in the gas mixture and that required for complete stoichiometric combustion, which was set to 1 for all argon admixtures.

### 2.2. Used Nanoparticles

The used nanoparticles are commercially available TiO_2_ (Aeroxide^®^ P25, Evonik) and CeO_2_ (NanoArc CE-0440, Alfa Aesar—now available as NanoArc CE-6440, Thermo Fisher Scientific, Waltham, MA, USA), which are available in powder form and as a suspension, respectively.

Aeroxide^®^ P25 is available as a powder with crystalline TiO_2_ with 85% anatase and 15% rutile modification. It is noteworthy that the more thermodynamically stable phase (rutile) is present to a much lesser extent despite the manufacturing conditions (flame hydrolysis). The rutile modification is concentrated on the particle surface and intensively interlocked with the anatase phase [30].

The used CeO_2_ is commercially available as a suspension with 25 w.-% CeO_2_ and 75 w.-% water, produced by Alfa Aesar, which is now part of Thermo Fisher Scientific. It is produced via a gas-phase plasma process through which the precursor is evaporated. After quenching the vapor, CeO_2_ nuclei form and grow to the desired nanoparticle size. These nanoparticles are then transferred to a suspension.

For the investigations, a suspension with demineralized water was prepared at a concentration of 4 g/L. This suspension was treated in an ultrasonic bath for one hour before starting the experiment and continuously stirred during the experiment to avoid agglomeration and aging as far as possible. The suspension was sprayed into the air by means of an atomizer (ATM220, Topas GmbH, Dresden, Germany) and added together with the premixed fuel gas at the inlet of the burner. The dosed mass flow of the suspension was determined at each test and was approximately 1.5 g/h.

### 2.3. Aerosol Sampling and Characterization

Above the burner, there is a tightly closing protective cylinder (length: 505 mm, inner diameter: 96 mm) made of quartz glass, which, on the one hand, prevents false air from leaking in and, on the other hand, offers a connection piece at different heights above the burner (HAB) for the connection of a sampling probe. In this study, the sampling probe, which is a 90° curved steel probe with a length of 172.5 mm and an inner diameter of 6 mm, was always installed in a HAB of approximately 450 mm. This corresponds to the highest possible measurement position. A dilution stage (VKL10E, Palas GmbH, Karlsruhe, Germany) upstream of the sampling probe diluted the number concentration of the aerosol to slow down coagulation processes. Additionally, it cooled down the aerosol sufficiently to be collected by the measuring device. The dilution stage has an input volume flow of 1.88 L/min (standard volume flow rate at 273.15 K and 1.01325 bar) and a dilution air volume flow of 20 L/min, resulting in a dilution factor of 11.6. The input flow rate was checked by a flow calibrator (Flow Calibrator 4148, TSI GmbH, Aachen, Germany), and the dilution air flow rate was set by a mass flow controller (EL-Flow, Bronkhorst, Ruurlo, The Netherlands). The particle size distribution was measured using a scanning mobility particle sizer (SMPS+C, Grimm, Ainring, Germany), which was operated with the M-DMA (Differential Mobility Analyzer, size range 5 to 350 nm), allowing mobility-equivalent diameters to be measured. The calibration of the mobility analyzer was performed with particle standards. Latex particles were used here because they are easy to handle and have a known density and spherical shape.

As a further measurement technique, the Electrical Low-Pressure Impactor (ELPI+, Dekati Ltd., Kangasala, Finland) was used. The ELPI consists of 15 impactor stages in a cascade setup. Before entering the cascade impactor, the particles are charged by passing a cylindrical tube corona charger. The charger efficiency is size-dependent and well-defined [31]. The mean aerodynamic diameter, which is the classifying physical quantity, ranges between 6 nm and 9.9 µm. The last stage (D50 = 6 nm) is a backup filter and collects all particles remaining in the gas flow. Each impactor stage is connected to an electrometer to measure the current of the impacted particles and to transform it into a number size distribution. It is possible to place foils or grids on each impactor stage to analyze the deposited particles via imaging techniques afterward.

For imaging, a transmission electron microscope TEM (EM910 Leo, Carl Zeiss Microscopy GmbH, Oberkochen, Germany) was used. The TEM grids were sampled in different ways depending on the research question. (a) To sample the original agglomerates without passing the flame, a TEM grid was installed in a filter housing at HAB ≈ 45 cm. (b) The sampling technique for imaging of aerosol particles that went through the flame included installing the TEM grid on the impaction plate of a low-pressure impactor where the complete aerosol is impacted. (c) For the comparison of volume equivalent diameter and aerodynamic diameter, TEM grids were installed on each ELPI impaction stage, where each stage corresponds to one specific aerodynamic diameter.

### 2.4. Equilibrium Temperature Calculations

For the comparison of the measured temperatures via CARS, equilibrium calculations were executed using ANSYS Chemkin [32] to provide values for the adiabatic flame temperatures with different flame stoichiometries and argon fractions. With the “equilibrium reactor”, it is possible to perform chemical and phase equilibrium calculations. GRImech 3.0 was used as a chemistry set for preprocessing, which includes thermodynamic data, transport data and gas-phase reactions for hydrocarbon combustion up to C_3_. The inlet gas temperature was set to 298 K, the pressure to 1 atm, and the problem type to “Constant Pressure Enthalpy”. Ethylene (C_2_H_4_) acted as the fuel and synthetic air (79% N_2_, 21% O_2_) as the oxidizer.

### 2.5. Temperature Measurement via CARS

As a non-intrusive laser diagnostic, coherent anti-Stokes Raman spectroscopy (CARS) has been well established as a reliable quantitative thermometry in various combustion environments [33]. CARS is nonlinear spectroscopy that provides spatially and temporally resolved temperature and species concentrations by probing molecular Raman shifts. Three coherent laser beams (pump, Stokes and probe) are focused and crossed in the region of interest, generating a CARS signal beam. The wavelengths of the three beams are chosen such that their interactions excite specific molecular ro-vibrational transitions of the target molecule. A broadband Stokes beam in combination with narrowband pump and probe beams (usually from the same laser source) is commonly employed to cover a wide range of vibrational and rotational transitions simultaneously. The N_2_ Q-branch is often adopted because of the abundance of N_2_ in the combustion environment. The resulting anti-Stokes signal beam carries the Raman spectra of N_2_. By comparing the experimentally obtained spectra to theoretical ones, the ro-vibrational temperature of N_2_ can be derived, which is assumed to be in equilibrium with the gaseous kinetic temperature in the measurement volume.

In this study, a frequency-doubled Nd:YAG laser (Quanta Ray Pro 290, Spectra Physics, Stahnsdorf, Germany) with a repetition rate of 10 Hz (λ = 532 nm, pulse duration ∼8 ns) was used to pump two dye lasers (Sirah Double Dye PrecisonScan, Sirah Lasertechnik, Grevenbroich, Germany). A narrowband dye laser was tuned to produce 591 nm, which provided the pump and probe beams (50% split). Furthermore, the broadband dye laser (Stokes beam) was set to peak around 685 nm with a bandwidth of about 10 nm. Compared with the often-used wavelengths of 532 nm and 607 nm, the choices of these specific wavelengths shift the CARS signal from 473 nm to 519 nm, hence avoiding signal interference due to laser-induced C_2_ emissions, a problem often encountered in sooting flames [34]. The lasers and the related optical components were mounted in a mobile container as detailed in [35]. To obtain a high-intensity CARS signal while maintaining high spatial resolution, a folded BOXCARS configuration [34] was used to achieve the necessary phase matching required for generating coherent CARS signal beams. Outside the laser container, the three laser beams (pump, Stokes and probe) were relayed by a series of high-reflectivity mirrors to the measurement position. An achromatic focal lens (f = 250 mm) was used to focus the three beams on the probe volume in the center plane of the burner. Prior to each measurement, a removable beam splitter was placed in front of the measurement position to deflect about 2% of the beams to a beam profiling camera (WinCamD, DataRay, Redding, CA, USA). The camera was placed at the focal plane of the three beams and was used to assist in fine adjustments of the beam overlaps. Spatial resolution was determined to be <0.1 mm in diameter (using the beam profiling camera) and about 1.5 mm (L95%) in the laser beam propagating direction using the non-resonant CARS signal from a thin quartz plate placed at the measurement position. At the crossing point of the three laser beams, the 519 nm CARS anti-Stokes signal beam was generated in room air or flame environments, which traveled coherently in the same direction as the other laser beams. It was captured by a plano-convex lens (f = 350) and directed into a spectrometer (THR 1000, Jobin-Yvon ISA Instruments, Longjumeau, France) via an optical fiber and a series of focal lenses. The spectrometer was equipped with an 1800 lines/mm diffraction grating and an entrance slit set to 50 μm. A bandpass filter centered at 520 nm (ET520/20 m, Chroma, Bellows Falls, VT, USA) was placed in front of the entrance slit to eliminate signal interferences from pump lasers. The CARS signal was imaged onto an intensified charge-coupled device (ICCD) camera (PI-MAX Gen III, 1340 × 700 pixels, Princeton Instruments, Krailing, Germany). The images were binned on-chip to 335 superpixels along the spectral axis and 1 superpixel in the vertical direction (i.e., 2D image converted into a 1D spectrum) to enhance the signal-to-noise ratio. For this investigation, each spectrum was obtained with an on-CCD accumulation of 300 single shots to further improve the signal level. Single-shot measurements were also carried out at selected positions for comparison and to characterize local fluctuations. The optics were mounted on translation stages so that different axial and radial locations in the flame could be probed.

Premixed C_2_H_4_–air–argon flames at various equivalence ratios and argon dilutions were measured in this study. All stable operating conditions examined are listed in Table 2. A radial symmetry was assumed for the stable flame, but asymmetric behavior was observed for certain conditions deviating from the reference flame (0% argon, Φ = 1). For cases other than the reference flame, only x positions were varied with r fixed at 0.

Prior to fitting the experimentally obtained spectra, the pixel axis needed to be converted to a wavelength (spectral) axis. For this, emission from a mercury–argon lamp (Avalight-Cal-Ar, Avantes, Apeldoorn, The Netherlands) was coupled into the spectrometer using the same fiber optic used for the CARS signal. In addition, a tungsten lamp was used to create a flat background profile to correct for any wavelength-dependent quantum yield (signal intensity) of the spectrometer as well as the camera.

Each CARS spectrum was accompanied by a background spectrum that was acquired right after the CARS spectrum and by blocking the laser beams. During the measurement campaign, pure argon flow was used regularly to obtain the spectral profile of the broadband Stokes beam (based on the non-resonant background), which directly affects the spectral intensity distribution of the CARS signal. In the end, each CARS spectrum prior to a fitting procedure was (1) subtracted by the background noise, (2) corrected for nonuniformities in the Stokes profile and (3) converted to a wavelength (wavenumber) axis.

For spectral fitting, a theoretical CARS spectrum was first generated and then convoluted with the laser profile (assumed Gaussian) and the slit function of the spectrometer. The slit function of the spectrometer was obtained by fitting a CARS spectrum taken in room air with known temperature with an asymmetric Voigt function [36]. The pump/probe laser linewidth was assumed to be 0.2 cm^−1^, as specified by the manufacturer. The exact value of the laser linewidth was found to exert negligible influence on the fitted temperature. Experimental spectra were then fitted iteratively against theoretical ones using a CARS-fitting routine “CARSpy” [37]. The routine was constructed to incorporate various well-known processes in the CARS process, including (but not limited to) the cross-coherence convolution of CARS susceptibility with pump/probe laser profiles, collision narrowing at moderate to high number density conditions (i.e., low temperature and/or high pressure) and Doppler broadening at high-temperature conditions [36,38]. Additionally, chemical equilibrium was assumed to calculate the local composition (for non-resonant background estimation) using the fitted temperature, and this was also included in the iterative fitting process. Since the mechanism used (GRI 3.0) is unreliable at low temperatures, the initial mixture composition was assumed to be intact until T = 1200 K. For the least-square fit, temperature was the only variable parameter in the theoretic model to minimize the fitting dependencies on inter-parameter correlations (e.g., among temperature, N_2_ mole fraction and non-resonant background).

## 3. Results

The Results Section is divided into two parts. Firstly, it shows a comparison of temperature measurement and equilibrium calculations. Secondly, it shows particle size distributions and TEM analyses of the used nanoparticles and comparisons of different measurement techniques.

### 3.1. Flame Temperatures

**Figure 2:**
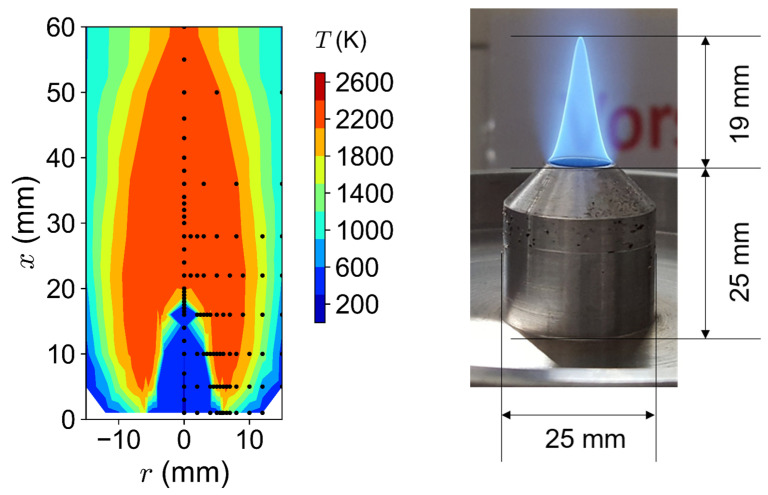
Left side: 2D temperature distribution reconstructed from local measurements for Φ = 1 and v_cold_ = 230 cm/s without argon. The black dots represent the measurement positions. Right side: an image of the burner nozzle with stabilized flame (0% Ar, Φ = 1) and respective geometric dimensions.

We measured the temperature profile of the Bunsen-type burner with CARS and compared it to Chemkin calculations at equilibrium. The temperature measurement was carried out without adding particles to the flame. We investigated the influence of the equivalence ratio (Φ = 0.83/1.0/1.25) as well as the added argon fraction (0%/7.5%/10%/12.5%/15%/17.5%/20%) on the temperature. The 2D temperature distribution of the flame is shown on the left side in Figure 2. The black dots show the measurement positions and the values are mirrored radially, assuming symmetry. The visible flame contour is shown on the right side of Figure 2. The conical flame stabilizes directly on the burner nozzle. The CARS measurements cannot be performed at x = 0 mm because the burner nozzle would inhibit a signal. Therefore, the left side of Figure 2 implies that the flame is lifted a few millimeters above the burner nozzle, but this does not correspond to reality. The flame shows a thin flame front, also recognizable at the steep temperature gradient. For this flame (0% argon, Φ = 1), the visible flame length is approximately 20 mm. Within the protective glass housing, no sheath flow was used.

**Figure 3:**
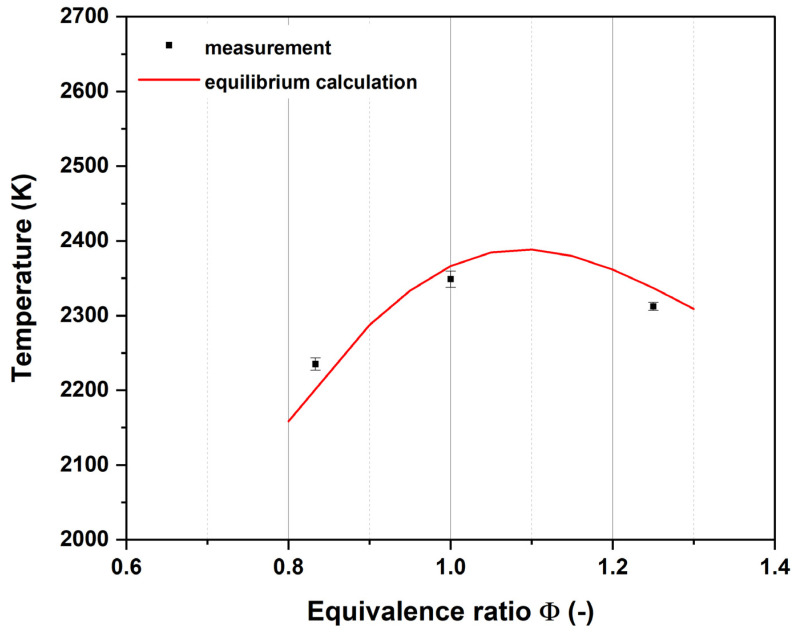
Comparison of measurement and equilibrium temperature for variation in the equivalence ratio without argon addition at r = 0 mm. The maximum temperature of the respective flame was used such that the axial measurement position is different (x = 36 mm for Φ = 0.833/x = 32 mm for Φ = 1/x = 30 mm for Φ = 1.25).

In Figure 3, the results show that the highest temperature was measured at an equivalence ratio of 1.0 with approximately 2350 K, as shown by the black squares. The calculation shows the maximum temperature at slightly under-stoichiometric conditions, at Φ = 1.1 with 2390 K, as shown by the red curve. The error bars represent the 95% uncertainty interval from the least-square fit of the experimental spectra.

**Figure 4:**
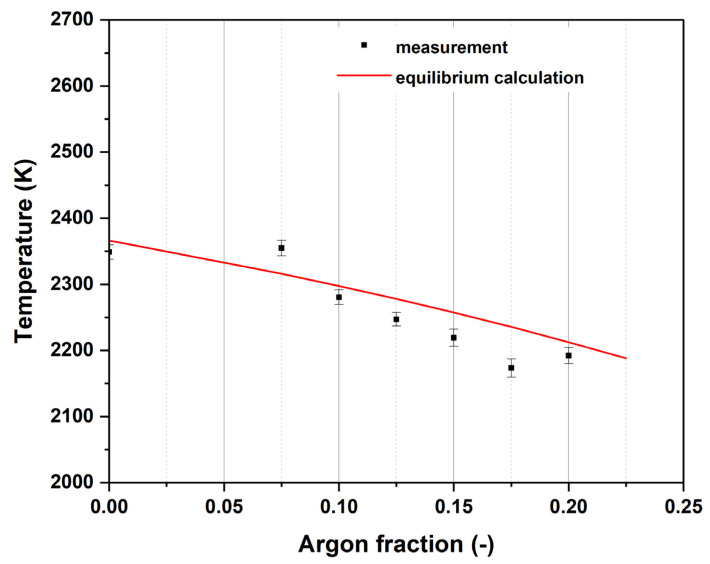
Comparison of measurement and equilibrium temperature for variation in the equivalence ratio without argon addition at r = 0 mm. The maximum temperature of the respective flame was used, such that the axial measurement position is different (x = 32 mm for Ar = 0, x = 40 mm for Ar = 0.075, x = 36 mm for Ar = 0.1, x = 40 mm for Ar = 0.125, x = 36 mm for Ar = 0.15, x = 37 mm for Ar = 0.175 and x = 38 mm for Ar = 0.2).

Figure 4 shows the temperature of the ethylene–air flame with different argon fractions. Starting with the highest temperature of approximately 2350 K at 0% argon addition, the values steadily decrease to 2220 K at a 20% argon fraction.

The accordance between the CARS measurement and the Chemkin equilibrium calculation is very good (see also Table 3), the deviation is at a maximum of 44 K, which corresponds to a deviation of 2% (at an argon fraction of 17.5%). For the discussion and the diagrams in the following sections, the calculated temperature values are used to avoid confusion due to small fluctuations in the measured values, which otherwise exhibit a clear downward trend with increasing argon volume fraction.

### 3.2. Aerosol and Particle Characterization

#### 3.2.1. Titania

**Figure 5:**
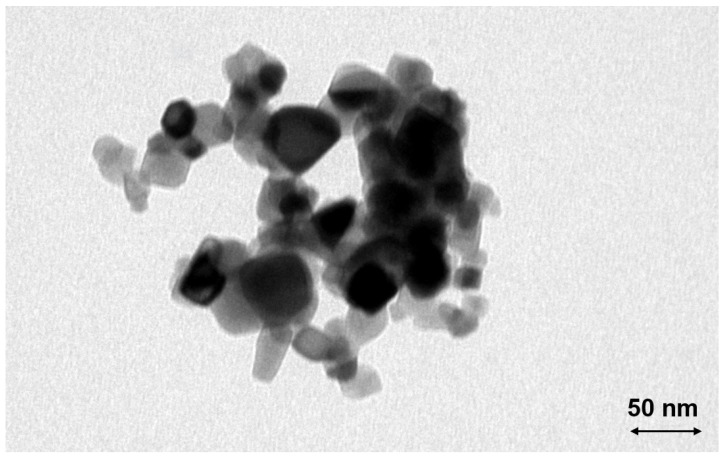
TEM image of the original TiO_2_ agglomerates within the suspension, sprayed via the atomizer and sampled on a grid in a filter housing approximately 45 cm above the burner (see TEM sampling case (a) in Section 2.3).

The measured TiO_2_ particles are primarily agglomerates with a mobility diameter of about 120 nm and a primary particle diameter of about 25 nm, according to the manufacturer. The representative TEM image (Figure 5) shows a large agglomerate, consisting of smaller primary particles. The particle and agglomerate sizes are in reasonable agreement with the manufacturer’s specifications.

To examine the possible aging of the suspension, a measurement of the thermally untreated suspension is carried out after each measurement with an appropriate flame setting, as shown in the following figure.

**Figure 6:**
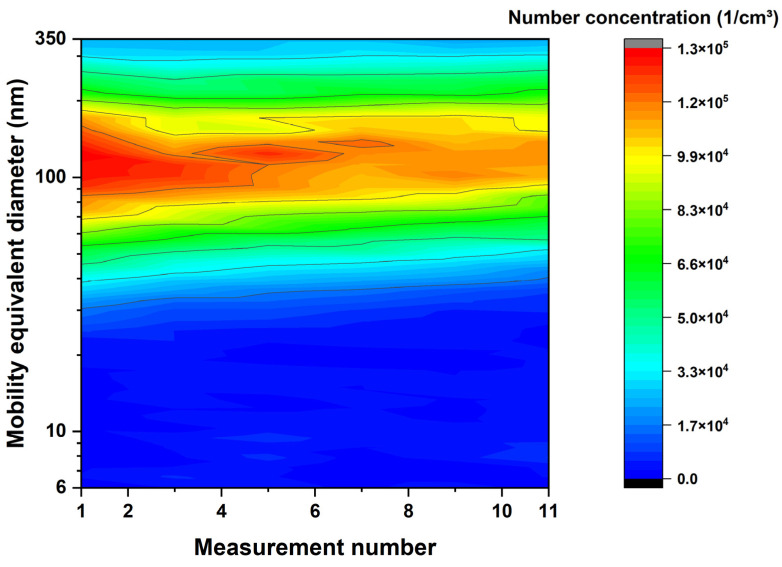
SMPS measurement of the titanium dioxide suspension without flame passage in order to monitor the stability of the suspension with respect to aging during the course of the test day.

Each measurement of the suspension consists of at least six scans. The time between these scans is approximately 5 min. The measurements of the suspension and the measurements of the suspension with ignited flame were carried out alternatingly so that the time between measurement #1 and measurement #11 was approximately 6 h. The average of the six or more scans for each measurement was used in Figure 6.

In the contour plot, the number concentration decreases slightly, and the particle diameter shifts to bigger sizes over time.

**Figure 7:**
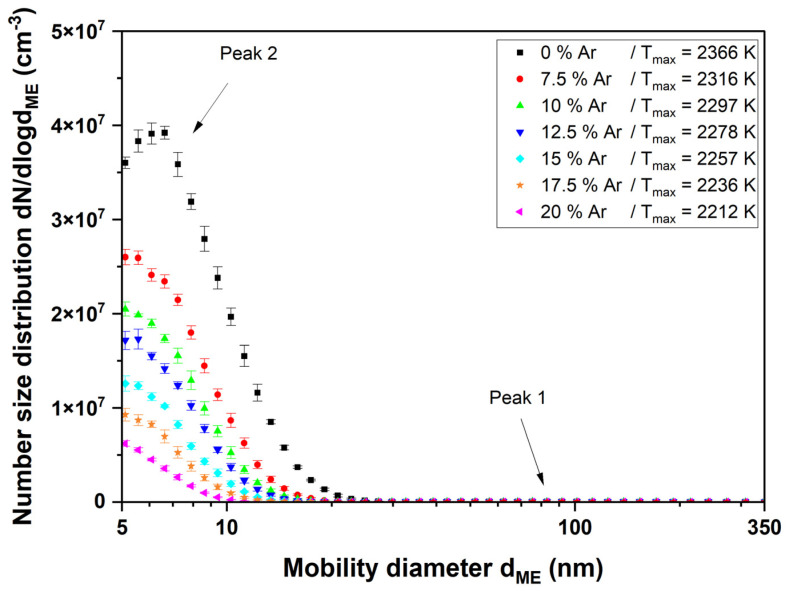
SMPS measurement of the titanium dioxide suspension with an ignited flame at Φ = 1. The argon admixture and thus temperature were varied to see the influence of the particle size distribution on peak 2.

When the combustion gases are switched on and the flame is ignited, the appearance of the particle size distribution changes considerably compared with the sprayed aerosol without combustion. In Figure 7, it is noticeable that a peak occurs at very small diameters of less than 10 nm (peak 2). With increasing argon content and thus decreasing temperature, this peak moves further to the left, while at the same time, its number concentration decreases. Another peak is not visible in this illustration because its number concentration is several orders of magnitude below the peak just described, so the scaling was adjusted in the following figure.

**Figure 8:**
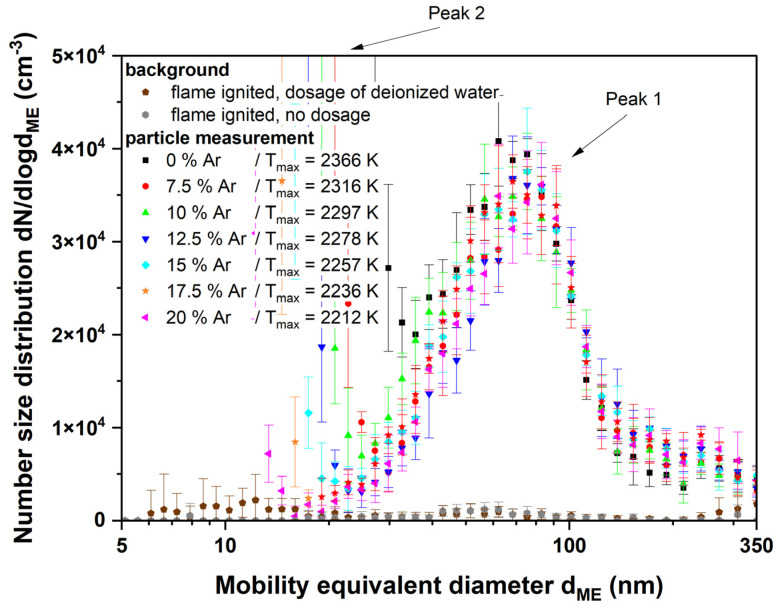
SMPS measurement of the titanium dioxide suspension with an ignited flame at Φ = 1. The argon admixture and thus temperature were varied to see the influence of the particle size distribution on peak 1.

In Figure 8, the “original” particle peak (peak 1) of the dosed particles can be seen, but it shifts to smaller particle sizes of approximately 80 nm, and its number concentration is decreased. Additionally, the figure shows the background signal from the flame itself (grey symbols) and the flame with sprayed deionized water (brown symbols).

**Figure 9:**
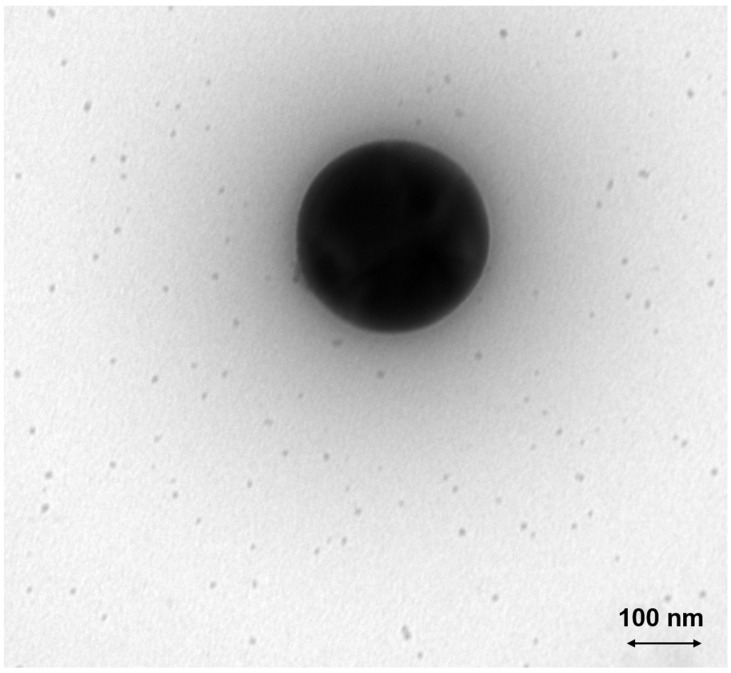
TEM image of TiO_2_ nanoparticles downstream of a flame with 0% Ar, Φ = 1 and T_max_ = 2366 K (see TEM sampling case (b) in Section 2.3).

To validate the results obtained by SMPS, a TEM grid was probed within a low-pressure impactor for the same conditions as described before, i.e., after the passage of a flame with T_max_ = 2366 K. A typical resulting TEM image is shown in Figure 9. A large spherical particle is present, as well as numerous small particles in the size range of 10 nm. The contrast of this large particle to the original agglomerate with primary particles of around 25 nm (see Figure 5) is obvious and will be discussed later.

For the following figure, an ELPI+ was integrated into the experimental setup instead of the SMPS. On the one hand, this also allows measuring the number size distribution online, but, on the other hand, the particles impact on the stages of the ELPI+ and can be used for subsequent analysis.

In this case, TEM grids were attached to the impactor stages, and the images of these grids were evaluated with respect to the number and size of the present particles.

For this purpose, one must consider that the ELPI+, in contrast to the SMPS, determines the aerodynamic diameter of the particles. Thus, in the simplest case of spherical particles, if we want to compare the geometric diameter with the aerodynamic diameter, the density of the particles must be considered.

In principle, the effective density of nanoparticles and their agglomerates cannot be explicitly defined. ENM may have internal voids that can significantly affect the value of the density in a simple comparison of mass and volume. However, since the TEM image in Figure 9 shows nearly perfectly spherical particles, the material (bulk) density is used here. Since the manufacturer’s specification states that P25 titania consists of 80% anatase (ρ_anatase_ = 3.9 g/cm^3^) and 20% rutile (ρ_rutile_ = 4.24 g/m^3^), the weighed density of 3.95 g/cm^3^ was used for the calculations.

**Figure 10:**
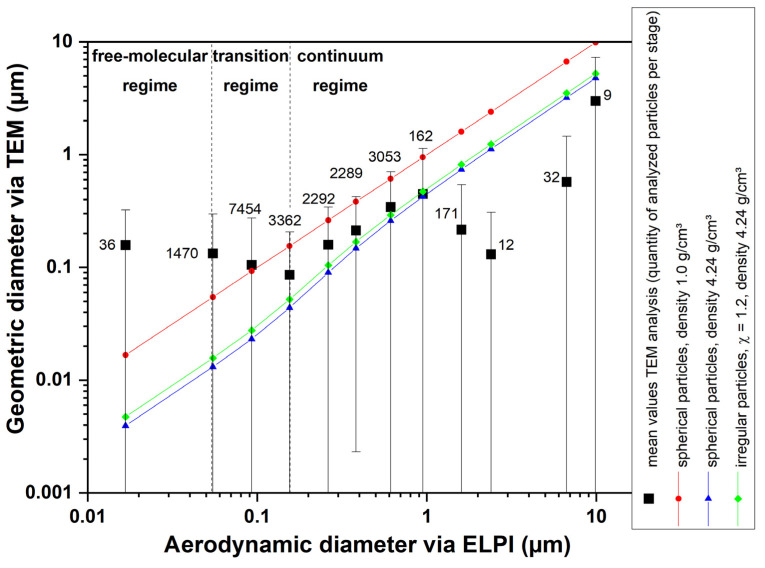
Comparative image analysis of TEM grids that were placed on different impactor stages of the ELPI+. The samples were taken downstream of a flame with 17.5% argon and Φ = 1 (see TEM sampling case (c) in Section 2.3).

Figure 10 shows a comparison of the aerodynamic diameter that is associated with the ELPI stages and the geometric diameter of the analyzed particles that were sampled on TEM grids at different ELPI stages. If the particles are spherical and the density is 1 g/cm^3^, the aerodynamic diameter equals the geometric diameter (red dots/red line). If the density is higher, the aerodynamic diameter increases compared with the geometric diameter (blue triangles/blue line). When the particles have a different shape than spheres, the change in the equivalent particle sizes is minor compared with the change in density (green diamonds/green line for a dynamic shape factor 1.2). The black rectangles show the mean diameter of the particles found on the TEM grid that was placed on the corresponding ELPI stage. The numbers show the count of particles that were analyzed per stage. A discussion of these results is provided in Section 4.

#### 3.2.2. Ceria

**Figure 11:**
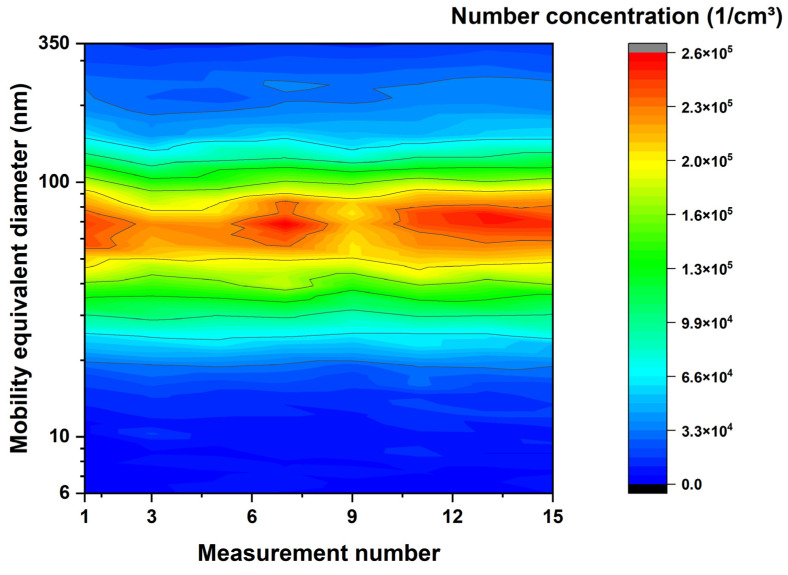
SMPS measurements of the cerium dioxide suspension without flame passage in order to monitor the stability of the suspension with respect to aging during the course of the test day.

For the contour plot (Figure 11), the suspension was measured alternatingly to the measurements with an ignited flame. Each measurement consists of at least five scans, whereby each scan took approximately five minutes. The time between the first and the last measurement of the suspension was approximately six hours. The contour plot of the ceria suspension shows a stable aerosol during the test day with a mean particle size of 65 nm.

**Figure 12:**
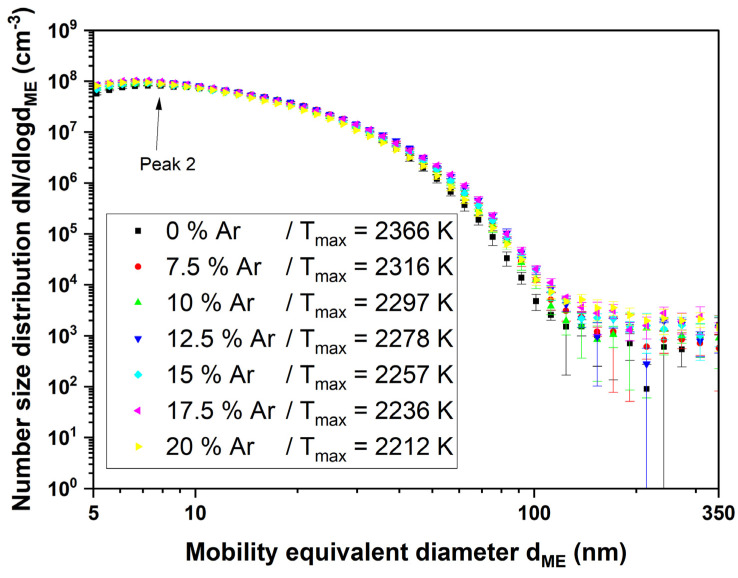
SMPS measurement of the cerium dioxide suspension with an ignited flame at Φ = 1. The argon admixture and thus temperature were varied to see their influence on the particle size distribution. The scale is double logarithmic to see the long tail of the particle size distribution at larger particle diameters.

**Figure 13:**
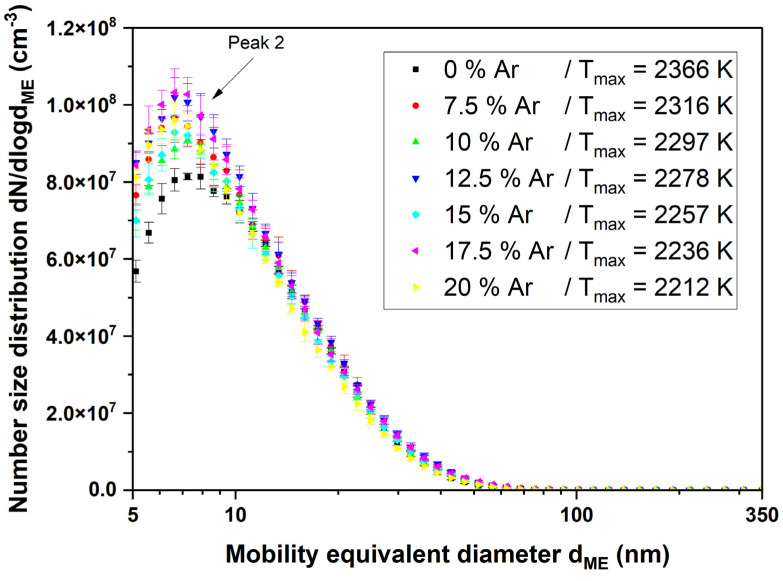
SMPS measurement of the cerium dioxide suspension with an ignited flame at Φ = 1. The argon admixture and thus temperature were varied to see their influence on the particle size distribution, especially on peak 2.

When the combustion gases are switched on and the flame is ignited (see Figure 12 and Figure 13), the appearance of the particle size distribution changes considerably. In contrast to the results with titania, the “original” peak cannot be seen anymore but the particle size distribution shows a long tail at larger particle sizes. A new peak occurs at very small diameters of less than 10 nm. Although there is no other peak in this size distribution, we call it “peak 2”, similar to the results of titania. Unlike titania, there seems to be no correlation between the argon fraction and the appearance of peak 2.

## 4. Discussion

### 4.1. Flame Temperatures

The measured temperatures at varying equivalence ratios (see Figure 2) are in good accordance with the equilibrium calculations. Adding argon to the gas mixture decreases the measured flame temperature (see Figure 3), which is also in excellent accordance with equilibrium calculations. These results appear to suggest negligible heat loss at the measurement locations on the burner/flame axis since the calculations were carried out assuming adiabatic conditions. This, however, might not be the case in the particle-laden flame. There are mainly two factors that could contribute to a deviation of flame temperature including the following: (1) CARS measurements had to be carried out in a relatively well-isolated chamber because of constraints imposed by the optical setup, which might have led to a smaller heat loss to ambient than in the case where the particles were introduced in open flames in a different laboratory environment. (2) The particles introduced into the flame themselves could also contribute to more radiative heat loss. However, since the total mass concentration of the added nanoparticles is very low for all the cases studied, their impact on flame temperature is considered minor. Overall, the potential systematic bias in the provided temperature values caused by the aforementioned factors is not expected to influence the interpretation of the measured particle behavior significantly.

### 4.2. Aerosol and Particle Characterization

#### 4.2.1. Comparison of Different Equivalent Diameters

Figure 10 shows a good agreement between the measured aerodynamic size (ELPI) and the geometric size (TEM) of the analyzed particles between 150 nm and 1 µm when spherical particle shape and density of 3.95 g/cm^3^ (blue triangles) is assumed (compare Equation (S2) in the Supplementary Materials). The agreement with spherical particles, the density of 3.95 g/cm^3^ and slightly irregular shape (χ = 1.2, corresponding to a compact agglomerate with four to five primary particles) (green diamonds) seems to be even better in the referred size range. Since we know that the particle shape is almost perfectly spherical based on the TEM images, the only plausible explanation is that the real density or effective density of the titania particles is lower than the calculated value of 3.95 g/cm^3^. It is possible that the ratio of rutile to anatase changed during annealing in the flame, which would decrease the density. The bulk density of anatase is 3.9 g/cm^3^; therefore, the overall density cannot be lower than this value. This minor density change between 3.95 g/cm^3^ and 3.9 g/cm^3^ would not explain the observed deviation. Another possibility of a lower effective density is if there are internal voids in the particles. Since the former agglomerates sintered to spheres, there can be internal voids, although there is no evidence on the TEM images. However, for titania, effective densities as low as 0.8 g/cm^3^ for slightly sintered agglomerates have been reported before [39].

In the aerodynamic size range below 150 nm, the measured geometric diameter is much larger than expected. The only explanation is the well-known “bounce-off” of particles from the above stages [39,40,41]. Thus, particles with larger diameters can penetrate to stages with smaller cut-off diameters that do not correspond to their actual aerodynamic diameter.

In the aerodynamic size range above 1 µm, the agreement between the aerodynamic diameter and geometric diameter is also not good. The main reason for this deviation is a lack of statistics because only very few particles could be analyzed in these ELPI stages.

#### 4.2.2. Titania

Figure 6 shows the contour plot of the sprayed TiO_2_ aerosol over the measurement number. The time between the first and the last measurement is approximately six hours and therefore covers the stability of the suspension over the course of the day. There is a slight increase in particle diameter, which is due to the agglomeration of the particles in the suspension. As part of this, the number concentration decreases slightly with each measurement. This minor altering of the suspension was tolerable because the only way to prevent it was to use stabilizers in the suspension, which would have other unwanted effects in the experiment.

The particle size distribution of the titania nanoparticles changes considerably after igniting the flame (see Figure 7 and Figure 8). The formerly large agglomerates at approximately 130 nm (peak 1) are still present, although slightly decreased in particle size. Additionally, a new particle peak (peak 2) forms at very small particle sizes around 10 nm. By comparing Figure 5 and Figure 9 it is obvious that the TEM images support the SMPS data. The former fractal-like agglomerates sintered into large spherical particles (250 nm in this specific image). Additionally, a lot of very small nanoparticles with diameters around 10 nm appear. The sintering or melting of the titania agglomerates decreases their mobility diameter, which can be seen by the slightly decreased mobility diameter of peak 1 in Figure 9.

Furthermore, the number concentration in peak 1 decreases compared with the initial aerosol. This can be explained by the fact that a new particle peak builds at small particle sizes with material from peak 1. The particle size of the newly formed particle peak is even below the primary particle size of the original agglomerates. Therefore, the fragmentation of agglomerates can be excluded as an explanation.

#### 4.2.3. Ceria

Figure 11 shows a very stable CeO_2_ aerosol during the test day. The stability is even better than the stability of the titania suspension (see Figure 6).

The appearance of the particle size distribution with ceria particles added to the flame is considerably different from the one with titania. We can also see the formation of a new particle peak (peak 2) at very small particle sizes, but the former peak 1 is not visible anymore (see Figure 12 and Figure 13).

Based on former studies by our group [17,19], we know that the formation of the new particle peak can be observed with considerably lower temperatures in the case of CeO_2_. The lower temperatures can be achieved by using another burner (McKenna burner), where the flame is stabilized on a sintered metal plate. This leads to a bigger heat loss compared with the Bunsen-type burner used in this study. For CeO_2_, the new particle peak can be observed with temperatures around 1475 K. For this condition, peak 1 (original particles) is still present, as is the case for the titania particles in the temperature range of this study (starting temperature < 2212 K). Considering physical properties like melting temperature and boiling temperature for both materials (ceria: T_m_ = 2773 K, T_b_ = 3873 K/titania: T_m_ = 2116 K, T_b_ = 3245 K), the observed effect cannot be explained by a “physical-only” explanation, namely, evaporation, nucleation and coagulation. For both materials, the maximum temperatures were far below their bulk-material boiling temperatures.

Since the maximum flame temperatures are below the melting temperature of ceria, enhanced vaporization of ceria in the chemically reacting flame is assumed

## 5. Conclusions

The approach in our study was based on the question of how nanoparticles behave during thermal treatment in the context of nano-enabled products that reached their end of life. Now we know that metal oxide nanoparticles form even smaller nanoparticles in high-temperature combustion environments. The starting temperature for this effect is material-dependent, which could be seen in the very different behavior of ceria and titania. Since the maximum flame temperatures are below the evaporation temperature of titania and ceria, a “physical-only” explanation, namely, vaporization, nucleation, coagulation and surface growth, is excluded. Therefore, a chemical reaction needs to be considered and investigated in future studies. This aspect, which has not been reported yet in the context of nano-enabled products, needs to be considered, concerning the possible release of nanoparticles during waste incineration processes. For titania, this might not be too relevant, since the starting temperature for the new peak formation is way higher than usual temperatures during waste incineration (1100 K to 1500 K, depending on the type of such incineration). However, ceria shows the formation of very small nanoparticles in the range of 1475 K, which can appear in technical incineration processes. Only two materials are considered here, whereby more relevant metal oxides are available and used on a large scale. Thus, their thermal behavior as well as the precipitation behavior of smaller nanoparticles in the flue gas cleaning system need to be evaluated in future studies.

## Figures and Tables

**Table 1:** Estimated production amounts of different nanomaterials in t/a.

Material	Hendren et al. [6]	Piccinno ^1^ et al. [9]	Piccinno ^1^ et al. [9]	EC [4]	Holden et al. [7]	Giese et al. [5]	Janković and Plata [8]
	USA	Worldwide	Europe	Worldwide	Worldwide	Worldwide	Worldwide
carbon black	-	-	-	9,600,000	>10,000,000	-	-
SiO_2_	-	5500 (55–55,000)	5500 (55–55,000)	1,500,000	>2,400,000	100,000–3,000,000	~1,000,000
TiO_2_	7800–38,000	3000 (550–5500)	550 (55–3000)	10,000	>30,000	-	~100,000
CeO_2_	35–700	55 ^2^ (5.5–550)	55 ^2^ (0.55–2800)	10,000	<10,000	1000–100,000	~1000
CNTs	55–1101	300 (55–550)	550 (180–550)	Several hundreds	250	-	~3300

^1^ Median value (25 to 75 percentiles in parentheses). ^2^ CeOx is listed in [9].

**Table 2:** Volume flows (T = 273.15 K, *p* = 1.013 bar) of the gases as a function of the desired argon admixture. The total volume flow is fixed, which leads to a constant cold gas velocity of 230 cm/s. For particle measurements, the settings with Φ = 1 were used.

Argon Fraction	Equivalence Ratio Φ	Volume Flow, Argon	Volume Flow, Ethylene	Volume Flow, Air	Volume Flow, Atomizer
(%)	(-)	(L/min)	(L/min)	(L/min)	(L/min)
0	0.83	0	0.60	9.24	1
0	1.25	0	0.87	8.97	1
0	1.0	0	0.71	9.13	1
7.5	1.0	0.81	0.66	8.37	1
10	1.0	1.08	0.64	8.12	1
12.5	1.0	1.35	0.62	7.86	1
15	1.0	1.63	0.60	7.61	1
17.5	1.0	1.90	0.58	7.36	1
20	1.0	2.17	0.57	7.10	1

**Table 3:** Comparison of calculated equilibrium temperature and measured temperature (max. value/different measurement positions) for a stoichiometric ethylene flame with a cold gas velocity of 230 cm/s and varying argon fractions.

Argon Fraction	T_eq_	T_CARS_
(%)	(K)	(K)
0	2366	2349
7.5	2316	2355
10	2297	2280
12.5	2278	2247
15	2257	2219
17.5	2236	2173
20	2212	2192

## Data Availability

The data are contained within this article and the Supplementary Materials.

## Supplementary Materials

The following supporting information can be downloaded at https://www.mdpi.com/article/10.3390/nano14121047/s1, Section S1. Comparison of different measurement techniques and their equivalent diameters, containing S1.1 Volume equivalent diameter, S1.2 Aerodynamic diameter and S1.3 Mobility equivalent diameter; Section S2. Experimental CARS spectra, containing Figure S1. Axial and radial measurement positions relative to the geometric center of the burner exit (r = 0 mm, x = 0 mm); Figure S2. Sample least-square fits of an experimental CARS spectrum, obtained at x = 32 mm and r = 0 mm, with 0% Ar and Φ = 1; Figure S3. Sample least-square fits of an experimental CARS spectrum, obtained at x = 38 mm and r = 0 mm, with 20% Ar and Φ = 1; Figure S4. Sample least-square fits of an experimental CARS spectrum, obtained at x = 37 mm and r = 0 mm, with 17.5% Ar and Φ = 1; Figure S5. Sample least-square fits of an experimental CARS spectrum, obtained at x = 36 mm and r = 0 mm, with 15% Ar and Φ = 1; Figure S6. Sample least-square fits of an experimental CARS spectrum, obtained at x = 40 mm and r = 0 mm, with 12.5% Ar and Φ = 1; Figure S7. Sample least-square fits of an experimental CARS spectrum, obtained at x = 36 mm and r = 0 mm, with 10% Ar and Φ = 1; Figure S8. Sample least-square fits of an experimental CARS spectrum, obtained at x = 40 mm and r = 0 mm, with 7.5% Ar and Φ = 1 and Figure S9. Sample least-square fits of an experimental CARS spectrum, obtained at x = 30 mm and r = 0 mm, with 0% Ar and Φ = 1.25.
