# Supplementary material for: Degradation and Recondensation of Metal Oxide Nanoparticles in Laminar Premixed Flames
Source: Nanomaterials (Basel). 2024 Jun 18;14(12):1047. doi: 10.3390/nano14121047 (PMC11206552; doi:10.3390/nano14121047)
Supplement: Supplementary file 1 [file nanomaterials-14-01047-s001.zip › nanomaterials-3053293-supplementary.pdf]

Supplementary Materials

Degradation and Recondensation of Metal Oxide Nanoparticles in Laminar Premixed Flames

Nadine May [1,*], Werner Baumann [1], Manuela Hauser [1], Zhiyao Yin [2], Klaus Peter Geigle [2] and Dieter Stapf [1]

[1] Institute for Technical Chemistry, Karlsruhe Institute of Technology, 76344 Eggenstein-Leopoldshafen, Germany; werner.baumann@kit.edu (W.B.); manuela.hauser@kit.edu (M.H.); dieter.stapf@kit.edu (D.S.)
[2] Institute of Combustion Technology, German Aerospace Center (DLR), Pfaffenwaldring 38-40, 70569 Stuttgart, Germany; zhiyao.yin@dlr.de (Z.Y.); klauspeter.geigle@dlr.de (K.P.G.)
* Correspondence: nadine.may@kit.edu

S1. Comparison of different measurement techniques and their equivalent diameters

The measurement techniques used (SMPS, ELPI, TEM image analysis) refer to different equivalent particle diameters since the actual dimension "length" cannot be measured on nanoscale. To understand the differences and how one equivalent diameter can be expressed in terms of another, they are explained in the following.

S1.1. Volume equivalent diameter

For the comparison of particle diameters that were observed in TEM analysis with other measurement techniques, the diameter of a spherical particle (d_{VE}) is used, which has the same projected area on the TEM image as the particle under consideration.

S1.2. Aerodynamic diameter

The aerodynamic diameter d_{AE} is defined as the diameter of a sphere with a density of 1 g/cm^3, which has the same terminal settling velocity as the particle under consideration. The terminal settling velocity v_{TS} is obtained by combining the equation of motion and Stokes law

$$v_{TS} = \frac{\rho_P \cdot d_{VE}^2 \cdot g}{18\mu} \cdot \frac{C(d_{VE})}{\chi}, \quad (1)$$

where ρ_P is the particle density, g the gravitational acceleration, μ the dynamic viscosity of the fluid, C(d) the Cunningham correction, and χ the dynamic shape factor (unity for spherical shape). Equating the terminal settling velocity of the "standard" particle (ρ_0 = 1 g/cm^3, χ = 1) and of the irregular particle leads to

$$d_{AE} = d_{VE}\sqrt{\frac{\rho_P}{\rho_0} \cdot \frac{C(d_{VE})}{C(d_{AE})} \cdot \frac{1}{\chi}}. \quad (2)$$

It is obvious that the aerodynamic diameter is increasing with particle density and decreasing with increasing dynamic shape factor. For standard density, the aerodynamic diameter is always smaller than the volume equivalent diameter if the particle shape is any different than a sphere (χ > 1).

S1.3. Mobility equivalent diameter

The SMPS uses the mobility of charged particles in an electric field to sort them by size or more accurately by mobility (which is also proportional to the number of charges). The conditions in a DMA are such that the terminal velocity of the charged particles in the electric field is in equilibrium with the drag force governed by Stokes, which leads to

$$Z = n \cdot e \cdot \frac{C(d_{VE})}{3\pi\mu d_{VE}\chi} \quad (3)$$

$$d_{ME} = d_{VE}\frac{\chi}{n} \cdot \frac{C(d_{ME})}{C(d_{VE})} \quad (4)$$

where Z is the mobility and e the charge of an electron. The mobility equivalent diameter is always bigger than the volume equivalent diameter with χ > 1.

Only in the simplest case, for standard density and spherical shape, all those equivalent diameters are equal. In any other case additional information about the particles is needed to compare the results of the different measurement techniques.

S2. Experimental CARS spectra

CARS measurements were carried out at various axial and radial positions relative to the geometric center of the burner exit, as plotted in Figure S1.

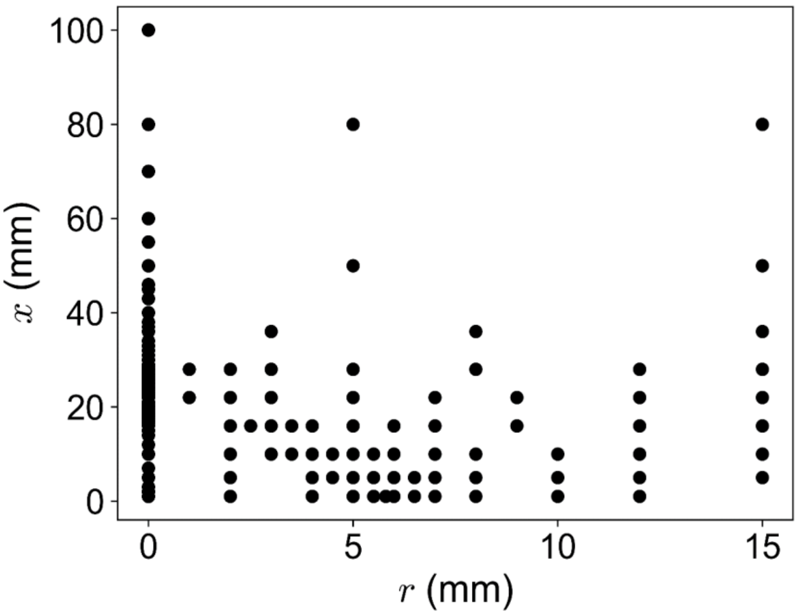

Figure S1. Axial and radial measurement positions relative to the geometric center of the burner exit (r = 0 mm, x= 0 mm).

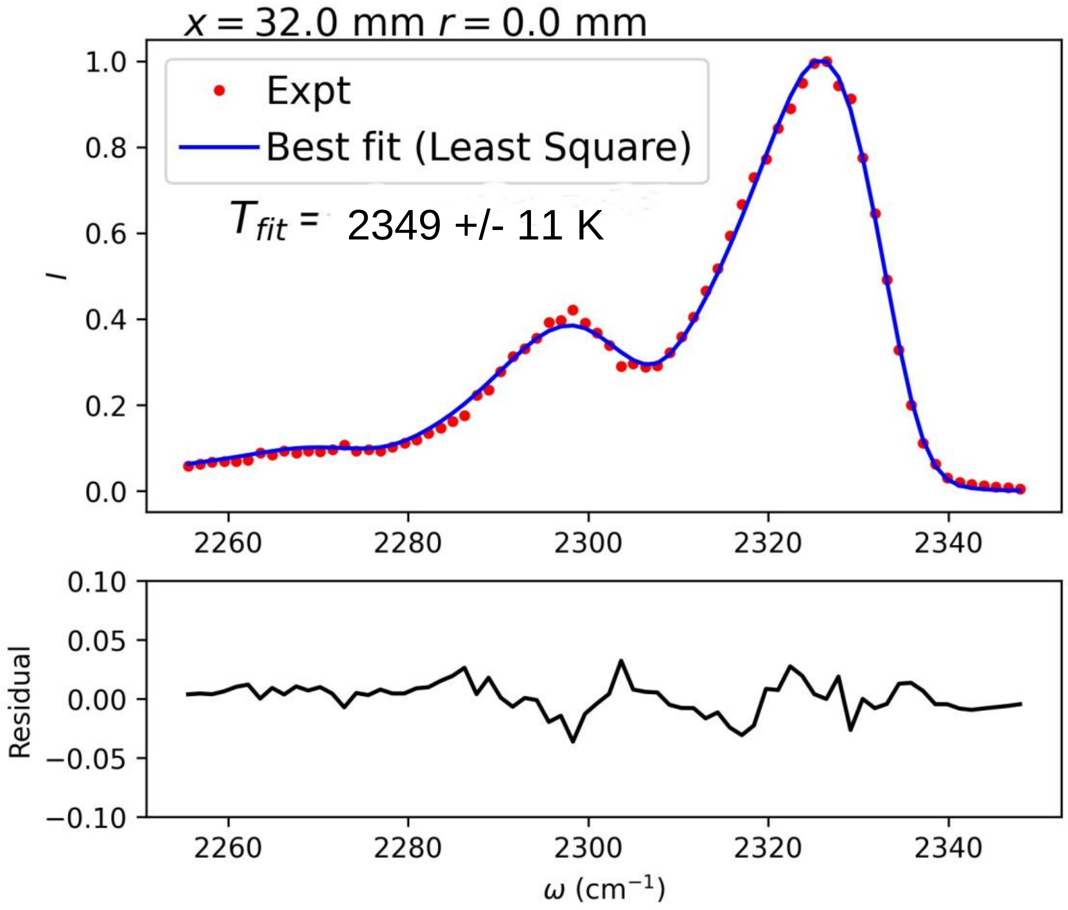

Figure S2. Least-square fit of the experimental CARS spectrum, obtained at x = 32 mm and r = 0 mm, with 0 % Ar and Φ = 1.

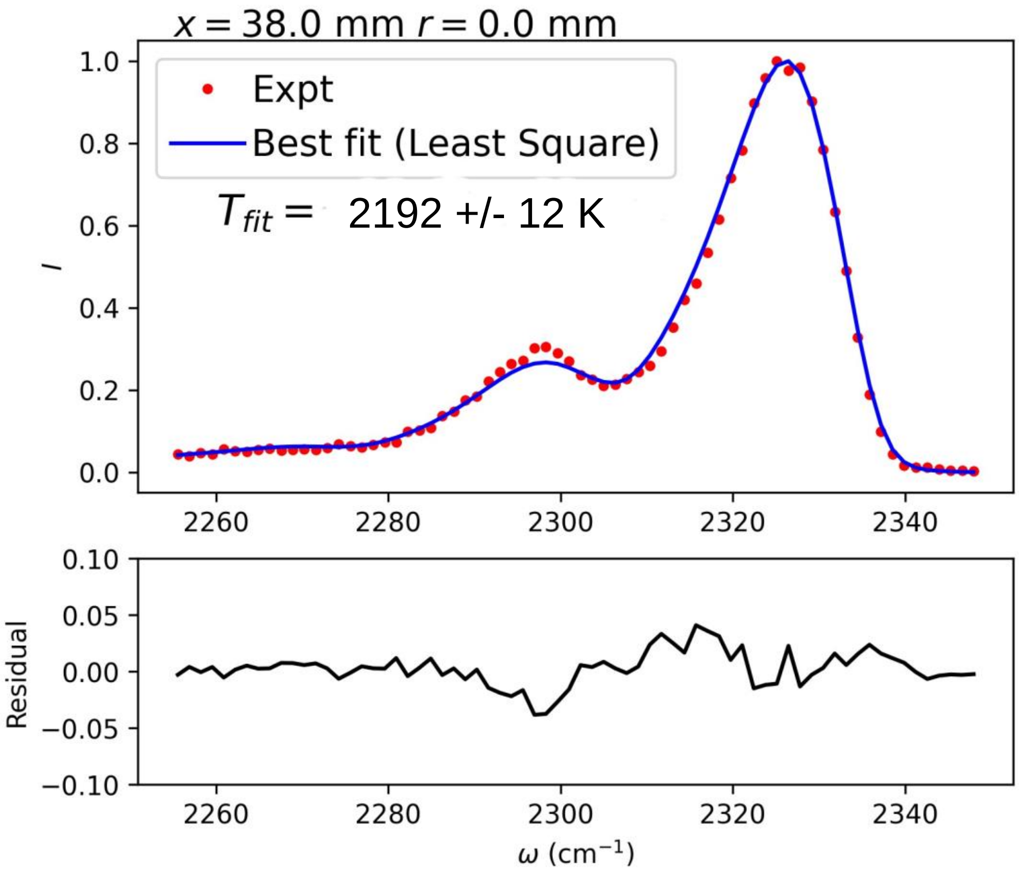

Figure S3. Least-square fit of the experimental CARS spectrum, obtained at x = 38 mm and r = 0 mm, with 20 % Ar and Φ = 1.

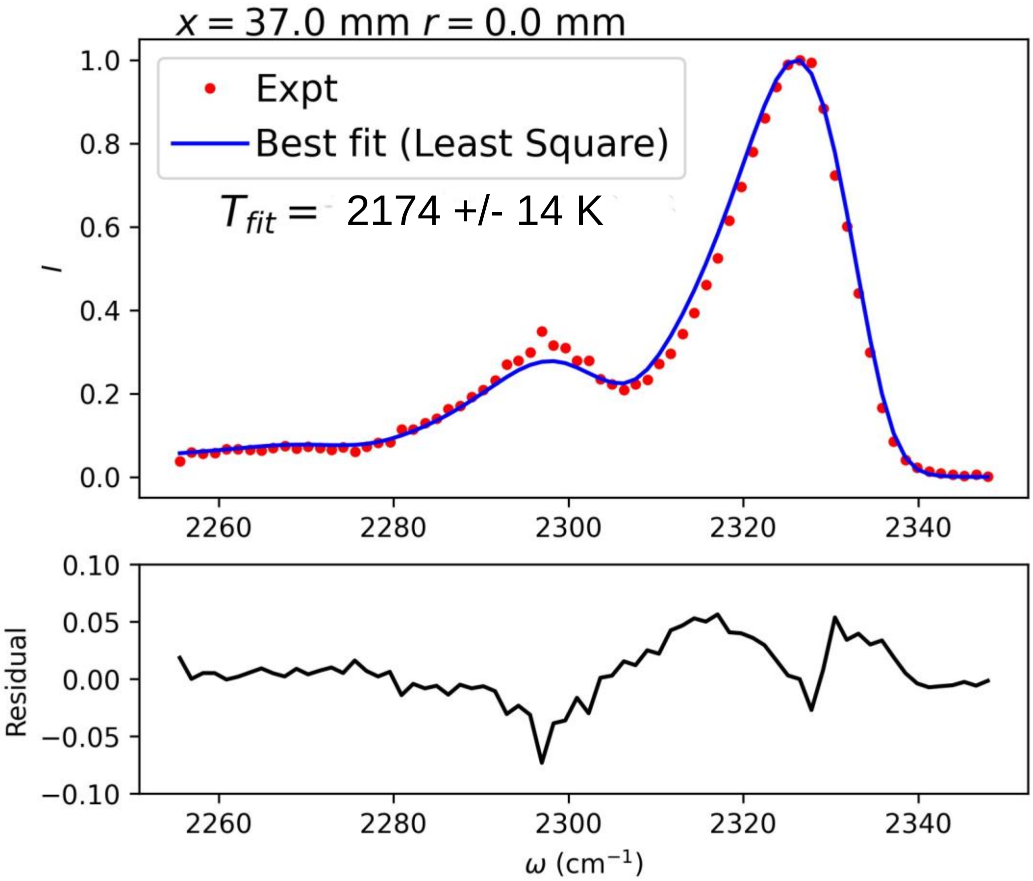

Figure S4. Least-square fit of the experimental CARS spectrum, obtained at x = 37 mm and r = 0 mm, with 17.5 % Ar and Φ = 1.

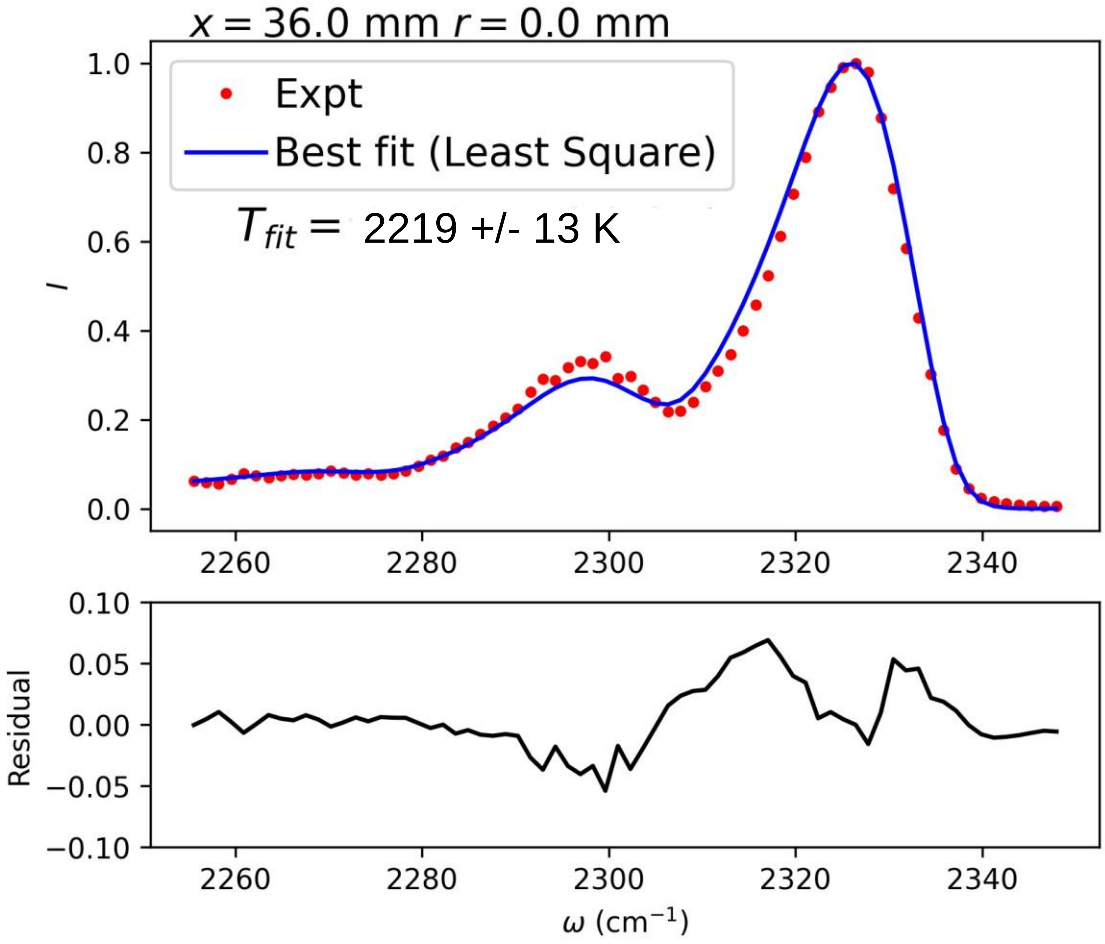

Figure S5. Least-square fit of the experimental CARS spectrum, obtained at x = 36 mm and r = 0 mm, with 15 % Ar and Φ = 1.

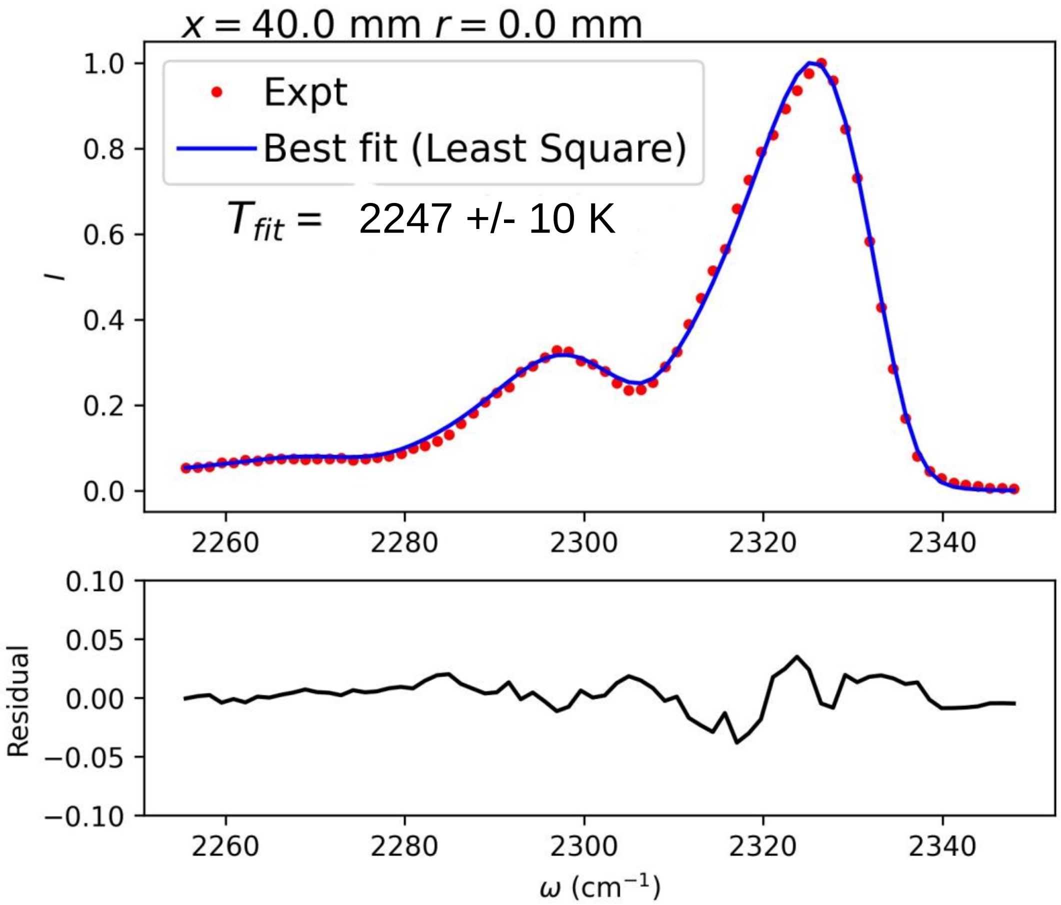

Figure S6. Least-square fit of the experimental CARS spectrum, obtained at x = 40 mm and r = 0 mm, with 12.5 % Ar and Φ = 1.

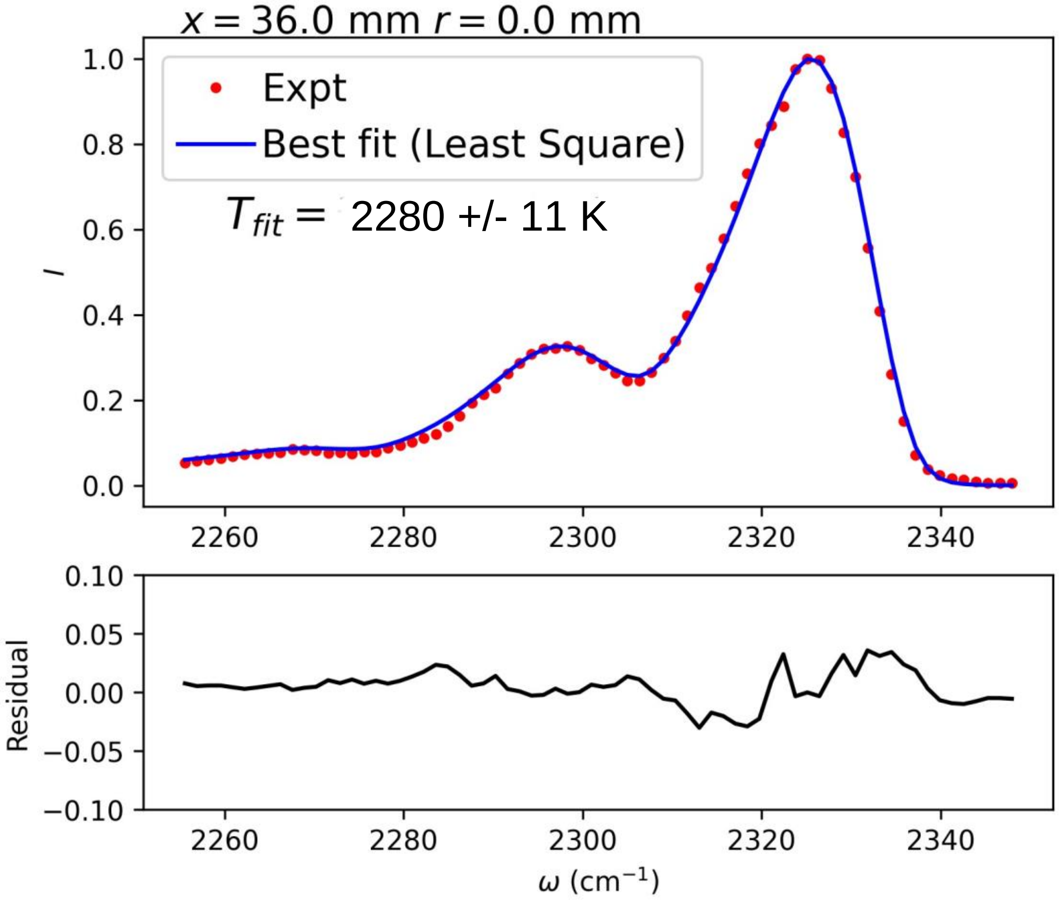

Figure S7. Least-square fit of the experimental CARS spectrum, obtained at x = 36 mm and r = 0 mm, with 10 % Ar and Φ = 1.

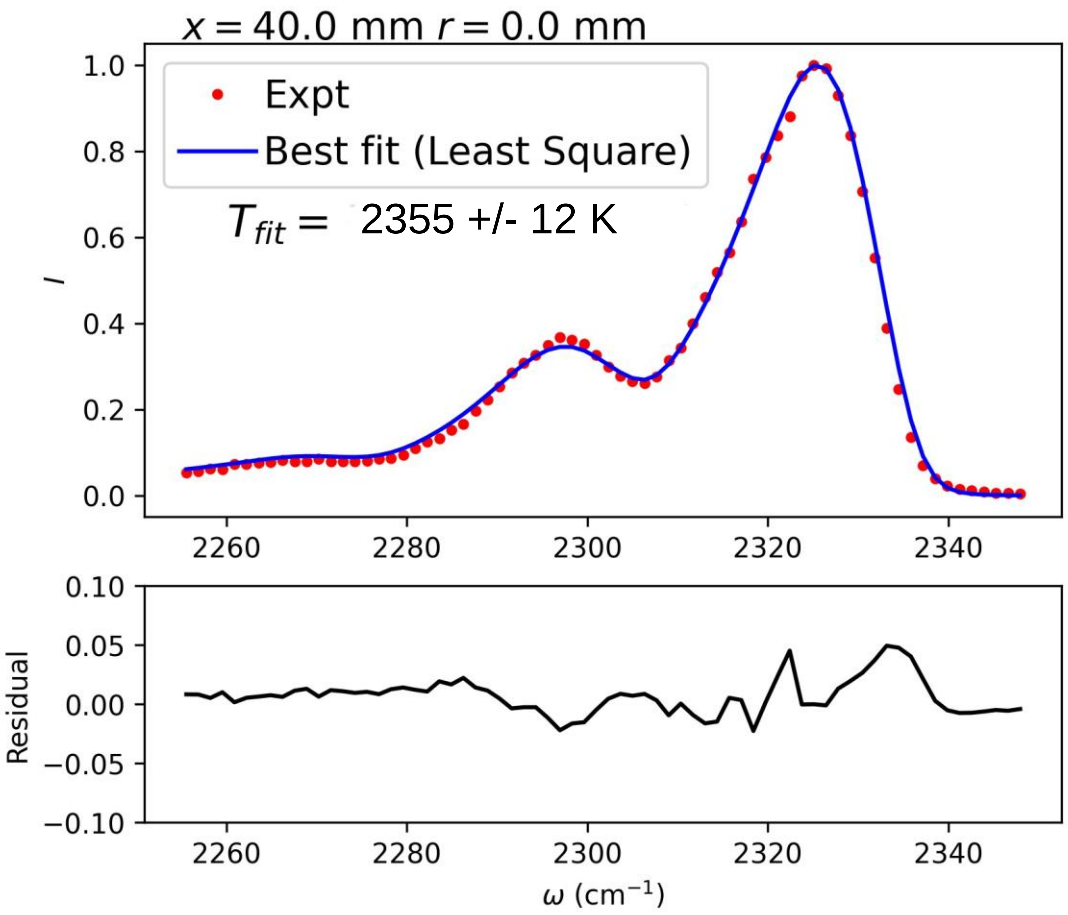

Figure S8. Least-square fit of the experimental CARS spectrum, obtained at x = 40 mm and r = 0 mm, with 7.5 % Ar and Φ = 1.

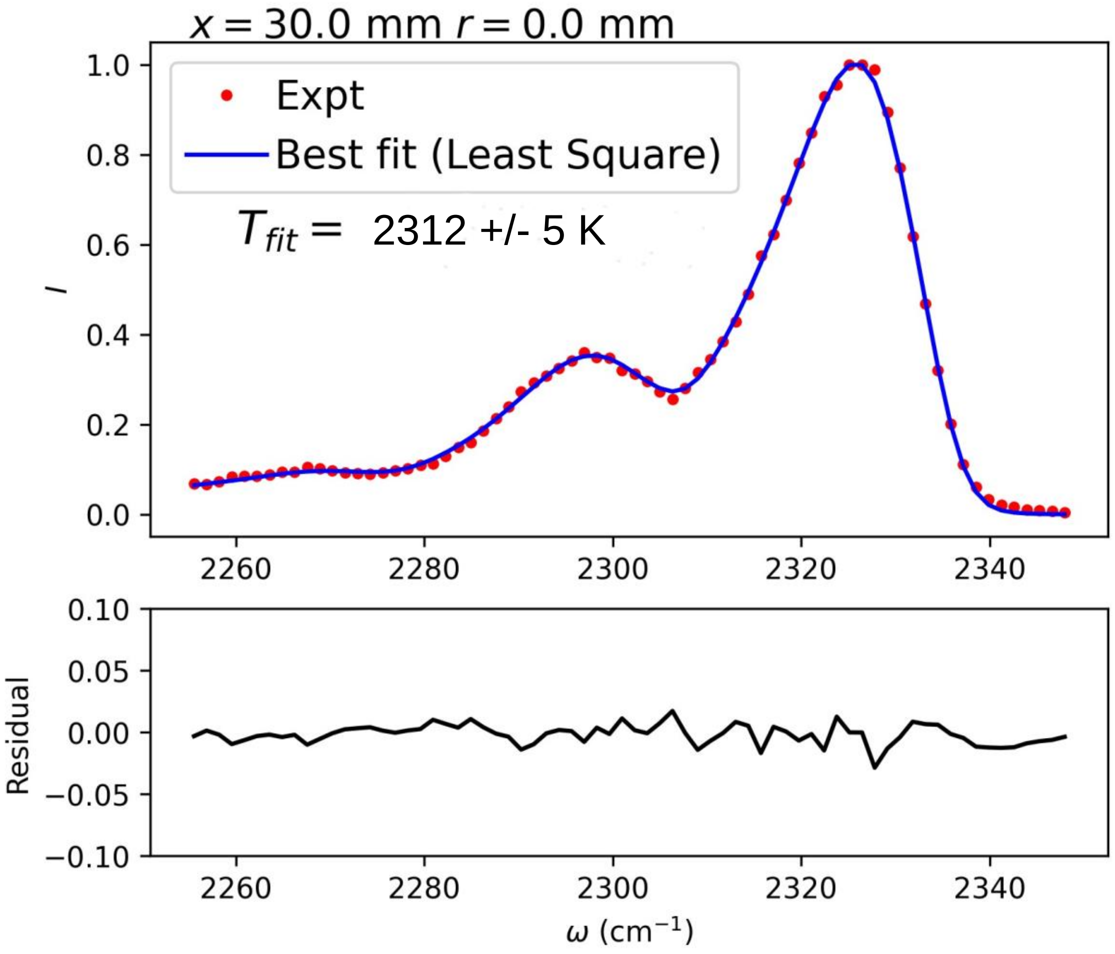

Figure S9. Least-square fit of the experimental CARS spectrum, obtained at x = 30 mm and r = 0 mm, with 0 % Ar and Φ = 1.25.
